# Evaluating Organizational Performance of Public Hospitals using the McKinsey 7-S Framework

**DOI:** 10.1186/s12913-021-07402-3

**Published:** 2022-01-02

**Authors:** Malgorzata Chmielewska, Jakub Stokwiszewski, Justyna Markowska, Tomasz Hermanowski

**Affiliations:** 1 Department of Forensic Pharmacy, Pharmacy Division, Medical University of Warsaw, 81, Żwirki i Wigury Str, 02-091 Warsaw, Poland; 2 National Institute of Public Health – National Institute of Hygiene, 24, Chocimska Str, 00-791 Warsaw, Poland

**Keywords:** Organizational performance, Model 7-S Framework Management, Public hospitals, Physicians

## Abstract

**Background:**

This study examined non-financial aspects of the organizational performance of public hospitals from the perspective of hospital physicians; the obtained results were analyzed to identify the necessary improvements in organizational performance.

**Methods:**

This was a cross-sectional study of multidisciplinary public hospitals on a group of 249 randomly selected physicians from 22 in-patient departments or clinics operating in the Warsaw region. The study data was collected using the structured World Health Organization questionnaires (to be filled out by respondents) assessing the hospital’s organizational performance variables qualified according to the McKinsey 7-S Framework.

Epidata software version 3.1 was used for data entry, and the analysis was carried out in the SPSS software, version 19. The results of the organization evaluation are presented in the McKinsey 7-S Framework diagram. Key elements of the performance factors were grouped into ‘stens’, and the sten values were expressed as arithmetic means. Normal distribution of the stens was validated with the Kolmogorov–Smirnov test. 95% confidence intervals were calculated. The significance of differences between the analyzed stens was compared with the paired Student t-test. The interdependence of the variables was determined using the Pearson’s correlation coefficient.

**Results:**

The results revealed a significant difference (p <0.05) in the respondents’ assessment of social (a mean score of 2.58) and technical (a mean score of 2.80) organizational aspects of the hospital operation. Scores for all variables were low. The social elements of an organization with the lowest score included ‘staff’, and in it the aspect – ‘efforts are made to inspire employees at the lowest levels of the organization’, ‘skills’ involving the learning style followed by the management/managerial staff, and ‘management style’ (average scores of 2.38, 2.56, 2.61, respectively).

**Conclusion:**

Consistently with the existing literature, social factors were shown to play a more significant role in the management and they therefore deserve careful attention and more recognition when identifying and improving the key aspects affecting the organizational performance of public hospitals. Technical elements (strategy, structure, system) are important, but were demonstrated to have limited effect on the organizational operations geared towards ensuring effective functioning of a public hospital.

## Background

Public health experts worldwide have been studying organizational performance of healthcare institutions, despite the inherent difficulties involved. There is a strong pressure to use quantitative indicators to assess organizational performance of healthcare organizations due to the increasing need for a more effective use of resources in the health sector. Stakeholders are also interested in these analyses as they hope to gain deep insights into the real-world use of public funds [1, 2].

In-patient treatment is extremely expensive – it accounts for up to 70% of the overall healthcare expenditure in some countries [3], and hence researchers turn their attention to the performance of hospitals. In highly developed countries, the primary research focus is on the operating theaters as the most cost-generating hospital units [4]. Today, improving hospital performance can be challenging [5].

In this paper, we seek to examine the factors that affect organizational performance of public hospitals using the McKinsey 7-S Framework against the background of the management process.

## Literature

### Organizational performance of public healthcare units

H. Emerson and H. Le Chatelier are considered to be the “founding fathers” of the concept of management by objectives, and their work was later extended by PF Drucker, who is given credits for introducing the notion of organizational performance to the theory of management [2]. His principles of performance remain valid to this day and are often referred to in the research literature. Emerson and Le Chatelier defined performance as doing the right things in the right way [6]. Likewise, J.A.F Stoner, R.E. Freeman, D.R. Gilbert argue that organizational performance is determine by organizational efficiency and effectiveness in achieving the right objectives and building good relations between an organization and its environment.

In this context, M. Bielski, who closely associated the concept of performance with the essence of the organization, distinguishes between two approaches to assessing performance [7]:

- goal-oriented – performance is determined by how successfully an organization achieves its objectives,
- systemic – performance is tantamount to the volume of savings measured by the relationship between the outcomes achieved and the expenditure incurred [6]. J. Campbell and Bielski both consider these approaches to be complementary and put forward a thesis about the multidimensionality of the category of performance. This makes room for various criteria, both numerical and descriptive, to be taken account in the assessment of performance.

The traditional approach to performance is based on financial measures. Consequently, profit is the economic measure most often used to assess performance. Considering the multidimensional nature of performance, it seems justified to adopt a critical approach to this model as being based on obsolete measures, without taking account of non-financial measures [8–10] related to the social aspects of performance management, e.g. human resources (human capital and intellectual assets) [8], which have so far been sidelined, perhaps because they are difficult to quantify. An assessment based solely on the outcome measures can be compared to “driving a car with the rear-view mirrors only, without seeing the road through the windshield” [7]; focusing on short-term performance while pushing long-term planning and innovation into the background [11].

From the perspective of public, non-profit organizations that pursue primarily social goals, the basic problem of measuring performance is to prioritize the assessment criteria [12]. According to Donald Snow, one of the basic criterion used for assessing performance organizations providing public services is the degree to which they fulfil their mission [13]. Jay Weerawarden and Gilian Sullivan Mort argue that creating greater social value for stakeholders is the underlying mission of public service organizations and is more important than profits [14]. Therefore, to measure the performance of non-profit organizations in quantitative terms only raises specific concerns as it does not allow for the assessment of non-economic outcomes.

Since the 1980s, performance indicators, mostly financial ones, have been increasingly monitored in profit-oriented sectors of the economy [15], while in public services, including the healthcare sector, non-financial measures have been brought to the fore [16].

### Measuring organizational performance in the social aspect – the concept of stakeholders

The relativity of performance measurements raises an important question: Who wants to improve performance or, in other words, from whose perspective is the performance considered? [17]. It is difficult to design a universal one-size-fits-all concept of organizational performance assessment that would be suitable for all stakeholders. Each group has its own goals, preferences and values, which means that the various dimensions and indicators that make up the overall performance would not be assessed in the same manner [18]. The main methodological principle in studies concerning the organizational culture is to examine an organization as a micro-community [19]. If an organization wants to act strategically, it should take into account the needs and expectations of stakeholders who are key to its growth and development. Therefore, in addition to financial analysis, it is also necessary to measure performance in the social aspect, which can be based on the concept of stakeholders. The stakeholder theory appears to be crucial in the efficient management of organizations and addresses the shortcomings of earlier theories of performance measurement largely relying on financial indicators [20]. The best known definition of stakeholders is the one developed by R. E. Freeman in 1984, defining a stakeholder is an entity or group of shareholders that has the power to impact an organization, whether directly or indirectly [21]. According to this theory, an organization is a network of connections between people or institutions. The theory of stakeholders highlights cooperation as the essence and foundation of the success of an organization [20]. The supporters of the stakeholder concept believe that it is important to identify and meet the objectives of stakeholders in order to safeguard the proper functioning and performance of an organization [7]. However, the map of stakeholders should be narrowed down to those who exhibit the most significantly impact on the organization, i.e. key stakeholders [22]. According to Frączkiewicz-Wronka et al., the key stakeholders are those without whom the organization would not exist [23]. In the social aspect, performance means implementing the organization’s social goals to a sufficient degree. The social goals are determined by how much individual groups of stakeholders are satisfied with the issues that concern them most, of the degree to which priority needs of individual stakeholders are fulfilled [24]. Moreover, Bryson points out that the success of an organization crucially depends on satisfying key stakeholders [20].

Managements can shape the decision-making process related to the performance of physicians. Even more so as there exists a simple relationship: if the executives work to meet the essential workplace-related needs of physicians, the healthcare professionals will care more about the reputation of the hospital they work in, and will be more likely to recognize and satisfy patient needs [25].

### Measuring the organizational performance of healthcare organizations – a hospital

Measuring performance in the healthcare sector at the microlevel can be applied to health service providers, including hospitals. Hospital performance can be assessed from several dimensions and based on a multiple of criteria. Based on the financial dimension, the evaluation could include profitability (the hospital's ability to generate profit from margin and assets), financial liquidity (the ability to timely fulfil the hospital's financial commitments), capital structure (to what extent the hospital uses debt service coverage and equity financing), operational performance (hospital's ability to convert various assets or liabilities into cash or sales), costs (labor, hospital expenses per bed, total expenses per bed, and operating expenses), revenues (e.g. patient's net revenue per bed, net revenue, patient’s net revenue per adjusted discharge, and admission) and the use of fixed assets, such as the occupancy of hospital beds) [26].

The non-financial dimension can be traceable to the assessment of measures such as efficiency (technical and allocative), productivity, and outcomes [8], providing insights about the financial performance not revealed by conventional financial measures. Technical efficiency is an assessment of the degree of use of resources, and in this case, the most common indicators are: the occupancy of hospital beds, the average hospitalization duration, and the amount of resources available per patient or service, e.g. number of medical staff, number of beds. Allocative efficiency refers to the way in which the existing resources re distributed, it determines the choice of health services, patients and disease entities to be financed. Productivity can be understood as the capacity productivity (average number of patients per bed per year), and the manpower productivity (full-time-equivalent employee productivity). Outcome indicators are also used, i.e. the therapeutic outcomes that translate into the quality of the services offered [8]. Apart from quantitative indicators, such as the mortality rate, nosocomial infection rates, average waiting time for a medical service, etc., performance assessments should also include qualitative measurement, e.g. the levels of patient satisfaction.

The research literature provides examples of studies assessing the behavioral dimension of hospital’s non-financial performance, in which the main emphasis is placed on the assessment of meeting the individual needs of hospital staff [27, 28]. It is also argued that among all hospital stakeholders, healthcare professionals should be particularly strongly involved in designing and shaping the assessments of how performance indicators are measured [29]. The widespread increase in the presence and involvement of clinicians in the management of health care organizations is also postulated as it believed to have a positive impact on, inter alia, the social performance of service providers [30, 31]. In this context, the social aspects of performance management is the strategic focus area for hospital management. The performance assessment provides key information about the effectiveness of management and the value delivered to stakeholders – it is an indicator at managerial level [32]. In order to operate in a strategic way, hospital should strive to meet demands and expectations of those stakeholders without whom it would not be able to operate [20].

The medical staff are the key stakeholders of a hospital, apart from patients and their families [33]. Organizational performance of a hospital in the social aspect – which corresponds to the fulfilment of social goals – can be assimilated with the degree of satisfaction of physicians with aspects they consider most important, expressed with appropriate quantitative and qualitative criteria [24].

Meeting the needs and goals of physicians was also shown to be an important driver of job satisfaction, correlated with the improvement of the quality of healthcare services and patient satisfaction. Hence, work satisfaction among physicians plays a key role in the management [34–36]. Many experts in human resource management agree that the level of job satisfaction among employees deserves proper recognition, and the WHO European Office for Integrated Health Care Services suggests that the focus on medical personnel and management should be included in measurements and evaluations of hospital achievements [37].

As a result, hospital performance can be examined based on feedback from physicians, using the concept of stakeholders, i.e. to examine whether the needs of this professional group are met.

### McKinsey 7-S Model of management by objectives

The McKinsey 7-S Framework is a research tool that takes into account the multidimensionality of an organization, i.e. the level of organization, team, and individual. It was designed by R. Waterman, T. Peters and J.R. Phillips working for the company McKinsey [38, 39]. (The Balanced Scored Card model is currently often referred to as the model of choice to examine the performance of healthcare organizations, accounting for both financial and non-financial aspects [8]. However, as the originators of the BSC Model, Dave Norton and Robert Kaplan, point out, the seventh “S,” shared values, are not explicitly recognized in the BSC model, although it complements the McKinsey Model [40].) The McKinsey 7-S Framework is based on the concept of management by objectives. It invokes the notion of organization’s ‘health’, an intangible category that incorporates the management method and the organizational culture [39]. The 7-S concept helps analyze the dysfunctions of the management process [38].

**Fig. 1:**
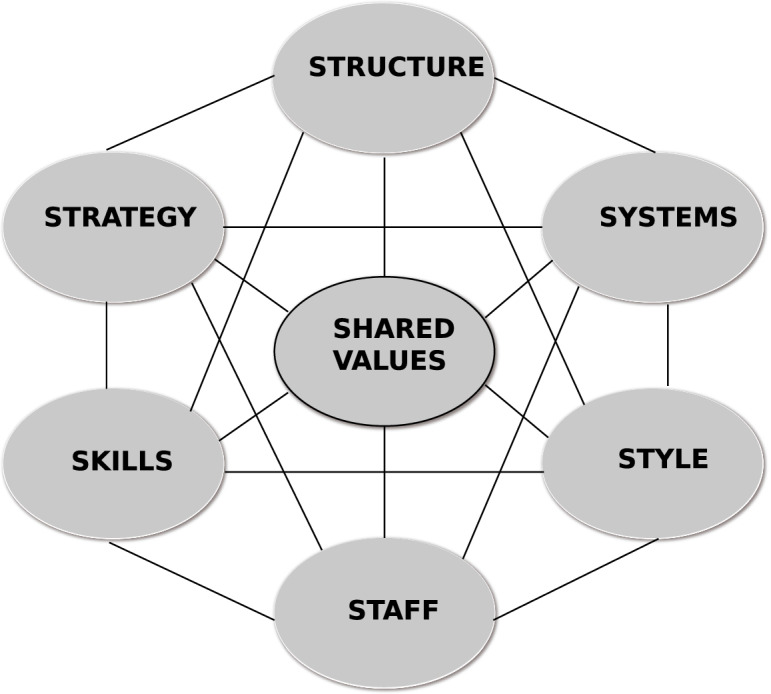
Organizational Performance Variables

The McKinsey model specifies seven factors (7-S) as the main variables that shape the organizational performance [38]: shared values, strategy, structure, system, staff, style, and skills. Organizational performance in the McKinsey’s model is a result of interactions among these variables. ‘Shared values’ is the focal point in this system (Fig. 1) [38].

The diagram is divided in two parts. The ‘hard’ part consists of structure, strategy, and system.

The structure is a model of functions and positions within an organization, often reflected in descriptions of hierarchies, authorizations, responsibilities, and functions.

The systems (procedures) are defined as processes in an organization within which individual employees and activities are managed, coordinated and directed to achieve the goals of the organization, e.g. the human resources system determines the manner of recruitment, promotion and transfer of employees.

The strategy means activities of an organization geared towards achieving goals and objectives, e.g. a strategy to improve the public health [38, 41].

The other elements represent the ‘soft’ part of the model.

‘Skills’ are defined as the total of individual capabilities of each employee in an organization, ‘staff’ are people employed by the organization who have different knowledge and experience, intelligence, ability and training; ‘style’ is a way of allocating rights and responsibilities within an organization; ‘shared values’ are beliefs, expectations and attitudes regarding work, organization, acceptable behavior, shared by the majority of employees, as well as any communication relating to the vision, mission and values that define an overall goal for all employees [38, 40, 41].

The ‘hard’ elements are related to the technical aspects of the organization, while the ‘soft’ ones represent its social part. In terms of management, soft factors are considered to be more prominent [38].

## Methods

### Main objective

Based on the literature and the proposed model, the following research hypothesis was formulated:

- H1: There is a relationship between organizational performance of a public hospital and the social (soft) elements of an organization (according to the classification of variables in the McKinsey Framework).

### Research tools and methods

The research tool employed in this study consisted of a validated World Health Organization (WHO) questionnaire used to examine the organization of public hospitals from the perspective of medical staff. 22 in-patient clinics/departments were randomly selected from among hospitals based in Warsaw, where a survey was conducted on a sample of professionally active physicians (n = 249). The physicians were randomly selected. Detailed selection of the study sample was described in a study by Chmielewska et al. [42]. Only general (multispecialty) public hospitals were included in the survey.

The hospital’s organizational performance factors were identified and evaluated on the basis of the McKinsey 7-S Framework. The survey consisted of 15 closed-ended questions concerning the ‘as-is’ and ‘should-be’ status of specific organizational features of a public hospital. The questions were divided into goal (mission) - the organization has a set of guiding beliefs, general objectives and values are set forth; strategy - the organization operates in a purposeful mode; structure - structures are designed based on work requirements; system - decisions are made based on location(s) of information, collaboration is rewarded, the organization is seen as an open system; management styles - managers assume individuals want more responsibility, rewards are balanced, communication is relatively open, conflict is managed, individuality and individuals are valued, management respects people; staff (support in career development) - an effort is made to inspire people and skills – there is a "learning" mode of management, feedback systems for assessing, regulating and responding to plans and actions are built in, in line with McKinsey's 7-S Model. The degree to which the organization’s performance needed to be changed was assessed on the basis of a comparison of the differences between ‘as-is’ and ‘should-be’ scores.

The hospital organization was assessed on a scale from 1 (the worst) to 6 (the best). Seven stens were calculated as a mean value of the survey questions. A mean value was also calculated for stens classified to either social and technical variable category. The mean status equals 3.5. Normal distribution of the stens was validated with the Kolmogorov–Smirnov test. 95% confidence intervals were calculated. The significance of differences between the analyzed stens was compared with the paired Student t-test. The interdependence of the variables was determined using the Pearson’s correlation coefficient. The significance level of 0.05 was adopted.

### Ethical considerations

This study was approved by the Ethics Committee of the Medical University of Warsaw (Approval No. AkBE/116/15). The researchers duly informed heads of hospital departments and medical doctors about the study. The contact details of the researchers and research information were included in the questionnaires. Participation in the study was voluntary, and the questionnaires were completed anonymously.

## Results

### Organizational characteristics of hospitals

**Fig. 2:**
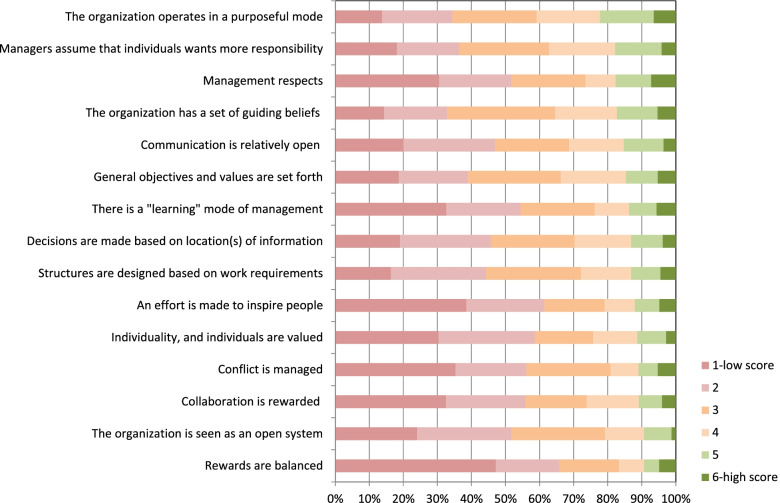
Characteristics of the hospital organization – assessment by physicians

Only over 20% of the surveyed physicians believed that their organization operates in a purposeful and goal-directed mode (a total of 5 and 6 scores (Figure 2)). Even fewer respondents (15% to 18%) felt that managers assume that individuals want to take on more responsibility and provide opportunities for them to do so, and that the managerial staff respect people, and the organization has set of guiding beliefs stated.

Only 14% of respondents believed that balanced rewarding is a strong point of the organization.

### Organizational performance of a hospital according to the McKinsey Framework

**Fig. 3:**
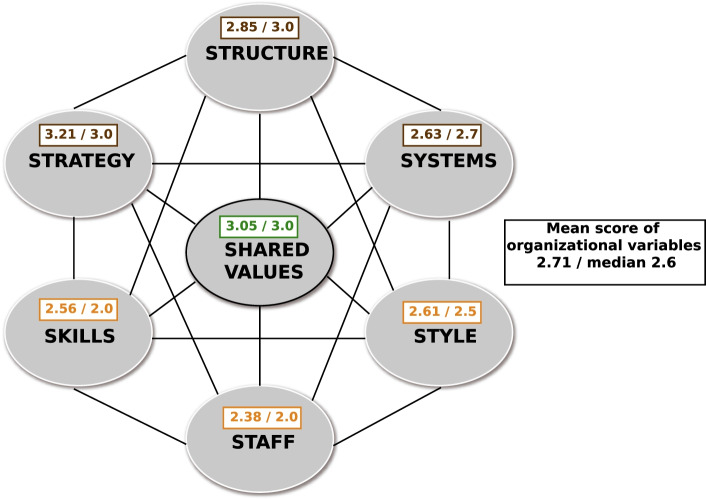
Variables of the McKinsey Framework, based on own research of hospital organizations

Strategy and shared value were ranked the highest in the survey consisting of questions assigned to the 7 organizational assessment criteria according to the McKinsey Framework. Staff was ranked the lowest. It is worth noting that all scores were relatively low or average. The mean score was 3.5 (Fig. 3).

With the mean score of 2.71 for organizational considerations (with the maximum score of 6) and the below-average scores for the remaining variables, it is safe to conclude that there is a widespread negative perception of the organizational performance of hospitals. All hospital organization variables scored low and very low in the survey conducted among the hospital physicians surveyed. This study confirms that the assumptions of the McKinsey Framework, i.e. the low score of individual variables that are strongly correlated with other variables and translate into reduced organizational performance of hospitals.

The examined organizational variables were divided into technical and social elements; the social (soft) aspects had statistically significantly (p <0.05) lower scores (a mean score of 2.58 (95% CI: 2.43-2.73)) compared to technical (hard) aspects with mean score of 2.80 (95% CI: 2.66-2.94). The scores of both technical and social elements were significantly below the mean of 3.5. The technical and social elements were found to be closely interrelated. The correlation coefficient equals 0.87. The ‘staff’, ‘skills’ and ‘style’ elements scored the lowest. The overall assessment of the organization is significantly reduced by the social aspects, which confirms the study hypothesis.

*How physicians evaluate: a) the as-is status of the hospital organization against b) the ‘should-be’ status (ideal organization).*

**Fig. 4:**
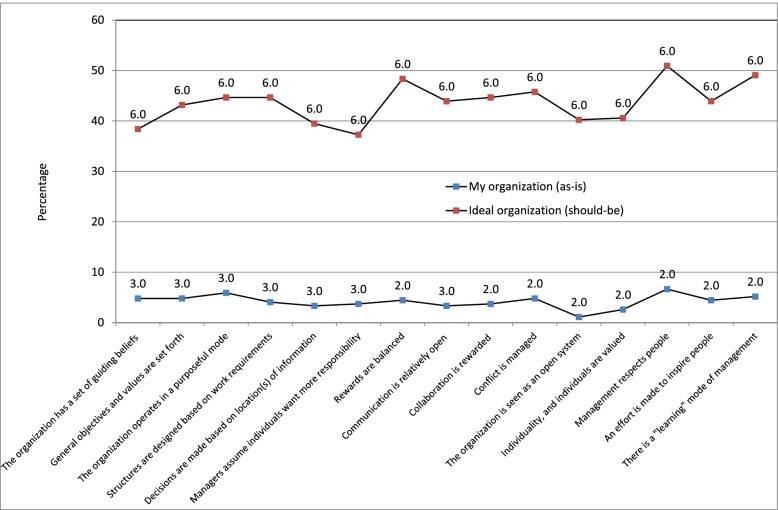
Characteristics of the hospital organization (as-is vs. should-be) - the percentage of physicians who assigned the maximum score for the analyzed aspects and the median value in the assessment by physicians

Unsurprisingly, according to the survey results, the score of the ‘should-be’ status (ideal organization) is significantly higher than the score of the ‘as-is’ status (Fig. 4). This is true for each of the 15 questions included in the survey. Percentages of physicians who gave maximum score to their own organization varies from 1.1% for question “The organization is seen as an open system” to 6.6% for “Management respects people”, while for should-be organization adequate percentages varies from 37.3% for “Managers assume individuals want more responsibility” to 50.1% for “Management respects people”. Medians for all questions are 6 for ideal organization but for as-is organization medians ear equal 2 or 3, therefore each examined aspect is expected to improve. In terms of the ‘should-be’ scores (ideal organization), both the median and percentages of maximum score for each of the 15 aspects are statistically significantly higher than the ‘as-is’ scores of the organization in which physicians actually work (p <0.05).

## Discussion

Recently, the organizational performance of hospitals has received widespread interest among researchers. With tight budgets and extensive restructuring, hospitals have been faced with new tasks and challenges as organizations. In addition to providing high-quality healthcare services, a hospital is expected to increase productivity despite budget restrictions, attract highly qualified staff and promote health. The modern perceptions about how a hospital should operate have inspired many models designed to assess its performance, unfortunately many of them focus only on isolated variables, and are therefore inclined to produce incorrect or unsatisfactory results [43]. According to literature, the operation of a hospital is multifaceted, it often exposes the challenges that hospital physicians are faced with, or unsolved problems, including issues with organizational climate, organizational structure, communication, and management [44]. According to E. Minvielle et al. [43], the hospital performance, or the ability to effectively treat patients, is more important than cost control, which is a challenge for a system focused on savings. It is of equal importance that a hospital is a place where members form groups with shared values.

The underlying assumptions, values [45], or, as Sikorski claims, patterns of thinking and actions necessary to achieve the goals of the organization create the essence of the organizational culture of a hospital [46]. A strong sense of the organization's ***mission*** is one of the key factors that may reflect organizational performance [47].

The following aspects: "The organization has a set of leading beliefs", “General goals and values have been presented" were rated at about 3, and “The organization operates in a purposeful manner" (strategy) scored 3.2. An ideal hospital in terms of organizational culture was rated 5.4 (‘should-be’ score). Different results were obtained in the study of organizational culture by MacKenzie. In a 1995 study on a group of 120 employees, including physicians working at 4 NHS health centers in the UK, respondents believed that their organization implements the following values: high quality of services, meeting goals and assumptions, caring for the satisfaction of beneficiaries, to complete effectively in the healthcare sector [48].

Here, the hospital ***structure*** was referred to as a ‘silo’. The system is considered ineffective in terms of its management mainly due to the separation between medical staff and the managerial staff, which translates into an unclear distribution of responsibilities and difficulties in quickly identifying and solving problems [49]. In this study, the hospital structure, which determines the specific division of competences and responsibilities, was also rated unfavorably by the surveyed physicians. A score of 2.8 is below average and the ‘should-be’ score was 5.4 (in an ideal organization). Similar findings were reported in studies by A. Montgomery et al. [50] conducted in eight European countries: Greece, Portugal, Croatia, Bulgaria, Romania, Turkey, Ireland, and Macedonia. Despite the diversity of the countries participating in this research project, the results of interviews and focus discussions among 153 physicians reveal a flawed organizational structure of hospitals, which results in high workload (administrative tasks, large number of patients, high responsibility for decisions regarding the treatment of many cases, shortages of human and material resources), and poor management, particularly the lack of cooperation between departments and the hospital administration. In a study conducted in the US [44], the respondents (n-816) also pointed to the existing deficiencies in the organizational structure, despite the general high level of satisfaction with the working conditions among hospital physicians. Many public sector organizations are deeply entrenched in centralized bureaucracy. This problem can be addressed by establishing autonomous hospital organizations and, for example, by delegating more responsibility for decisions to local administrative units, which inherently have a better insight into the work of hospital staff than distant central authorities [51]. In this study, the transfer of responsibility to lower organizational levels scored 3, which is an average rating compared to the ‘should-be’ score of 5.2. The sense of control over the work environment and participation in the decision-making were studied among 608 physicians from the United States. These aspects were shown to be the main predictors of mental well-being, satisfaction and professional commitment [52].

There is a scarcity of research on the possible relations between physicians’ involvement in administrative and political decision-making and the performance indicators of healthcare organizations. According to A. Kaissi [45], open and honest communication, especially as concerns the manager-doctor relationship, and joint commitment to decision-making bring in a number of benefits: increase in the number of patients, shorter hospital stay, and a higher hospital ranking.

Researchers found that empowering employees in the decision-making process significantly prevents the staff from adopting a cynical attitude towards the organization [53]. Hospital leaders should take particular care of activities facilitating the participation of physicians in decision-making concerning changes in the organization of a department or hospital [54]. It is also noteworthy that information and knowledge management is definitely of paramount importance for healthcare organizations [55]. According to scientific sources, there is a discrepancy in the management of information and knowledge between the head of the clinic and the hospital administration. In the healthcare sector, most problems arise because healthcare professionals are trained to care for and prioritize patients' needs [56]. In this study, the respondents gave a low score to the decision-making based on locations of information rather than roles in the hierarchy scored low. There is a considerable discrepancy between the ‘as-is’ and the ‘should-be’ score of 5.3 concerning this aspect. J.K. Barr [57] described the relationship between perceived participation in decision-making and three indirect organizational outcomes: satisfaction with the work of a doctor, the staff’s perceived consensus on daily activities, and attitudes towards patients. Physicians who reported greater involvement in organizational decisions were more satisfied with their work, perceived a better consensus among employees, and had better attitudes towards patients. A.M. Rotar et al. of 2016 [58] conducted a study under OECD in 188 hospitals based in 7 European countries, on a sample of 1,670 respondents from the Czech Republic, France, Germany, Poland, Portugal, Spain and Turkey; the results suggest that physicians at the department and hospital level are involved in formal decision making only to a limited extent.

The latest research underlines the need to explain the role of leadership in the medical community as there is a difference between the traditional work environment and the healthcare environment [59]. C. Ham [60] believes that the role of leadership is crucial in improving healthcare, in decision-making processes concerning the operation of hospital wards and all hospital patients. When the ***leadership style*** is consistent with the expectations of the subordinate staff, higher job satisfaction is recorded [61]. In this study, physicians assessed the hospital management, including its respect for hospital staff, inspiring and encouraging training, conflict management, communication, and fair rewarding. The average ‘as-is’ score for these aspects is 2.6 compared to the ‘should-be’ score of 5.5 for an ideal organization. It is also important to note that the highest ‘should-be’ scores were obtained for two management aspects, i.e. the respect of hospital managers towards staff and the learning mode. Likewise, in a study by H.M. Elarabi performed in 2014 [62] in a Libyan government hospital, most medical staff felt disregarded by the senior management. Physicians also complained about the lack of or unsatisfactory level of training. This has led to low job satisfaction and lower performance of the hospital staff. A study by B.S. Savič and M. Pagon [63] revealed that staff development was often neglected and hospitals failed to make use of the knowledge and experience of the healthcare professionals, which contributed to their limited commitment to work. Research has also found that leaders only partially fulfilled their responsibilities in terms of supporting and inspiring teamwork in hospitals. In general, organizations promote hierarchy.

Marina Kaarna [64] argues that the management and managers in healthcare institutions should learn how to express their appreciation for the commitment and efforts of medical staff. In this study, the ‘as-is’ scores for ***balanced rewarding*** were the lowest at 2.2, compared to the ‘should-be’ score of 5.4. In a study by J. Rosta [65] on a group of physicians in Germany (n - 1917), the ‘recognition for work’ as also considered unsatisfactory. According to the respondents, this factor was among the five lowest rated aspects of work. Similarly, in a study by S. Mackenzie [48], NHS physicians did not feel fairly recognized by the organization, despite they believed they were loyal and committed.

The issue of communication is perhaps one of the most important preconditions for improving motivation. It is most likely the key determinant of learning, coping and job satisfaction [66]. The aspect of ‘relatively open communication’ in this study has a low ‘as-is’ score of 2.4 against the ‘should-be’ score of 5.4. A comparable, or even less favorable conclusions about this issue were drawn from a study by B.S. Savič of 2008. It revealed a lack of good communication among the staff of Slovenian hospitals [63]. In this case, the surveyed healthcare professionals pointed out that autonomy and interpersonal communication were the most important factors responsible for job satisfaction and individual well-being. In a study by A.L. Tucker et al. of American hospitals, analyzing 1,732 medical errors made between 2004 and 2006, communication problems were shown to account for 16% of the most common errors of medical staff in hospitals [67]. Another US study on a group of 816 physicians also indicated that communication was the main area for improvement [44].

***Conflicts*** at various levels, relational, task-related and process-related, are an inherent part of the healthcare environment [45]. The effectiveness of cooperation largely depends on how conflicts are addressed and resolved. Interpersonal and process-related conflicts can lead to poor outcomes and lower job satisfaction, while task-related conflicts stimulate constructive criticism and allow to creatively challenge ideas and opinions, leading to better achievements. In a study by G. Mulvany [68], organizations that dealt effectively with conflicts had a high level of professional satisfaction. In this study, almost 60% of physicians surveyed had an unfavorable opinion on how conflicts are managed in their organization – no more than 2 on a scale of 1 to 6. The average ‘as-is’ score of conflict management is 2.4 (ranked third among the lowest rated aspects of organization), and the ‘should-be’ score is 5.3. In a 2003 study by S. Chaudhury, 72.7% of physicians ranked the inability to solve staff problems third most demotivating factor [69].

Research by S.L. Browning published in 2014 [25] provides insights into how to develop strong healthcare organizations by devoting more attention to the ***integration of physicians*** in the organizational structure, which delivers better results than paying for the treatment outcomes. B. Nunberg [70] also concluded that, because the effectiveness of promoting work motivation by paying for performance has not been confirmed, this method should not be given priority. In fact, paying for performance can have an unfavorable effect on the organizational culture by creating competition between physicians rather than a sense of collaboration to achieve a common goal-vision of the organization [25]. It was also found that physicians are more motivated by internal factors, and therefore strategies should be developed to facilitate physician involvement by facilitating career development and promoting collaboration among healthcare professionals. Over time, this will ensure greater job satisfaction, which in turn positively shapes the behavior of physicians, eliminates occupational burnout and medical errors, increases patient satisfaction, and improves clinical outcomes. In this study, the 'rewarding for collaboration' aspect scored 2.5, significantly below the average of 3.5. Physicians felt that a hospital should undertake efforts to stimulate cooperation (a ‘should-be’ score of 5.4). As shown in a study by B.S Savič in 2008 [63] conducted among healthcare professionals in 14 Slovenian hospitals, physicians also complaint about the lack of good teamwork. The study results suggest that the current work organization, level of teamwork and leadership model do not promote individual commitment to work, which may mean that employees are exposed to unfavorable pressure in their work environment. It was also found that those who do not work in a team show greater nervousness and lower job satisfaction compared to those who favor teamwork.

The final aspect of the organization evaluation was the use of the ***‘learning mode"*** by the hospital management. Mentoring is one of its important aspects, the essence of which is to facilitate the development of professional competences, especially among medical students and young graduates. Academic literature described mentoring programs in Sweden under which students receive the support of a doctor whose function is compared to a ‘conductor’ and not a teacher or an examiner in charge of assessing knowledge [71]. In this study, the ‘learning mode’ of management is among worst assessed features with a score of 2.6. However, it is also one of two characteristics with the highest ‘should-be’ score, i.e. in an ideal organization, it was given a score of 5.5.

In a qualitative study conducted in Stockholm using individual interviews (N = 12) of people participating in the program, the results showed that having a mentor provided a sense of security and was a ‘space of freedom’ in addition to the study curriculum. It offered more hope for the future and greater motivation; it helped introduce students to the new community and identify as physicians. S. Kalen et al. argue that individual mentoring can create favorable conditions for the development of professional competences that are not included in medical curricula, such as reflective skills, emotional competences and a sense of belonging to a community [72]. A study of H.M. Elarabi [62] analyzing low job satisfaction among physicians demonstrated that there is a lack of training programs and an absence of plans to increase the competence of hospital staff.

## Strengths and Limitation of the Study

The strengths of this study include random selection and relatively robust sample size coupled with the use of the WHO questionnaire and the relevant theories that frame the study and help to explain the results. The results of the present study and the results of research conducted in highly developed and developing countries were compared to confirm that the healthcare systems faced similar complex problems worldwide. The results of this study also confirm that more research needs to be conducted, including in hospitals operating outside large metropolitan areas. The use of bivariate tests of significance rather than multivariate models can be counted as a study limitation as it does not allow for the inclusion of relevant control variables.

## Conclusions

When identifying and improving essential aspects affecting the organizational performance of public hospitals, more attention needs to be paid to social factors as they were shown to play a more significant role in the hospital management. This conclusion is consistent with the existing literature and validates the study hypothesis. ‘Hard’ elements are undeniably important aspects of an organization, however, they determine the performance of management to a limited extent only, i.e. organizational activities and decisions leading to the achievement of the intended goals. In efforts to improve hospital performance, the method proposed illustrates the importance of a prospective study of hospital organization, in which the satisfaction of the key stakeholders is taken into account, to supplement the most common, yet inadequate methods of retrospectively analyzing hospital performance based solely on financial indicators.

With the proposed methodology, by processing input data concerning the organizational performance in a McKinsey 7-S Framework diagram, the hospital operation can be more effectively diagnosed to produce a more comprehensive assessment of its organizational performance, compared to relying on financial results as the sole performance measure.

The study contributes to the body of knowledge concerning a practical approach to hospital management, and points to the value of non-financial aspects in increasing the (organizational) performance of hospitals from the perspective of hospital physicians as the main internal stakeholders.

## Data Availability

All data can be requested from the corresponding author.

## Abbreviations

NHS: National Health Service
OECD: Organization for Economic Co-operation and Development
WHO: World Health Organization
